# Lentivirus-mediated shRNA Targeting CNN2 Inhibits Hepatocarcinoma *in Vitro* and *in Vivo*: Erratum

**DOI:** 10.7150/ijms.114964

**Published:** 2025-05-15

**Authors:** Xueqing Kang, Feng Wang, Xiuwan Lan, Xiaolong Li, Shunxin Zheng, Zhilue Lv, Yuan Zhuang, Yongxiang Zhao, Sufang Zhou

**Affiliations:** 1 Department of Biochemistry and Molecular Biology, Guangxi Medical University, 22 Shuangyong Road, Nanning 530021, the Guangxi Zhuang Autonomous Region, China;; 2 Guangxi Key Laboratory of Biological Targeting Diagnosis and Therapy Research, Guangxi Medical University, 22 Shuangyong Road, Nanning 530021, the Guangxi Zhuang Autonomous Region, China.

When reviewing the previous work, we identified an inadvertent error in the originally published version of the article—specifically, the immunohistochemical image for AKT in Figure 6A was mistakenly used. We sincerely apologize for this error. Figure 6A should be corrected as follows. The authors confirm that the correction made in this erratum does not affect the original conclusions.

## Figures and Tables

**Figure 6:**
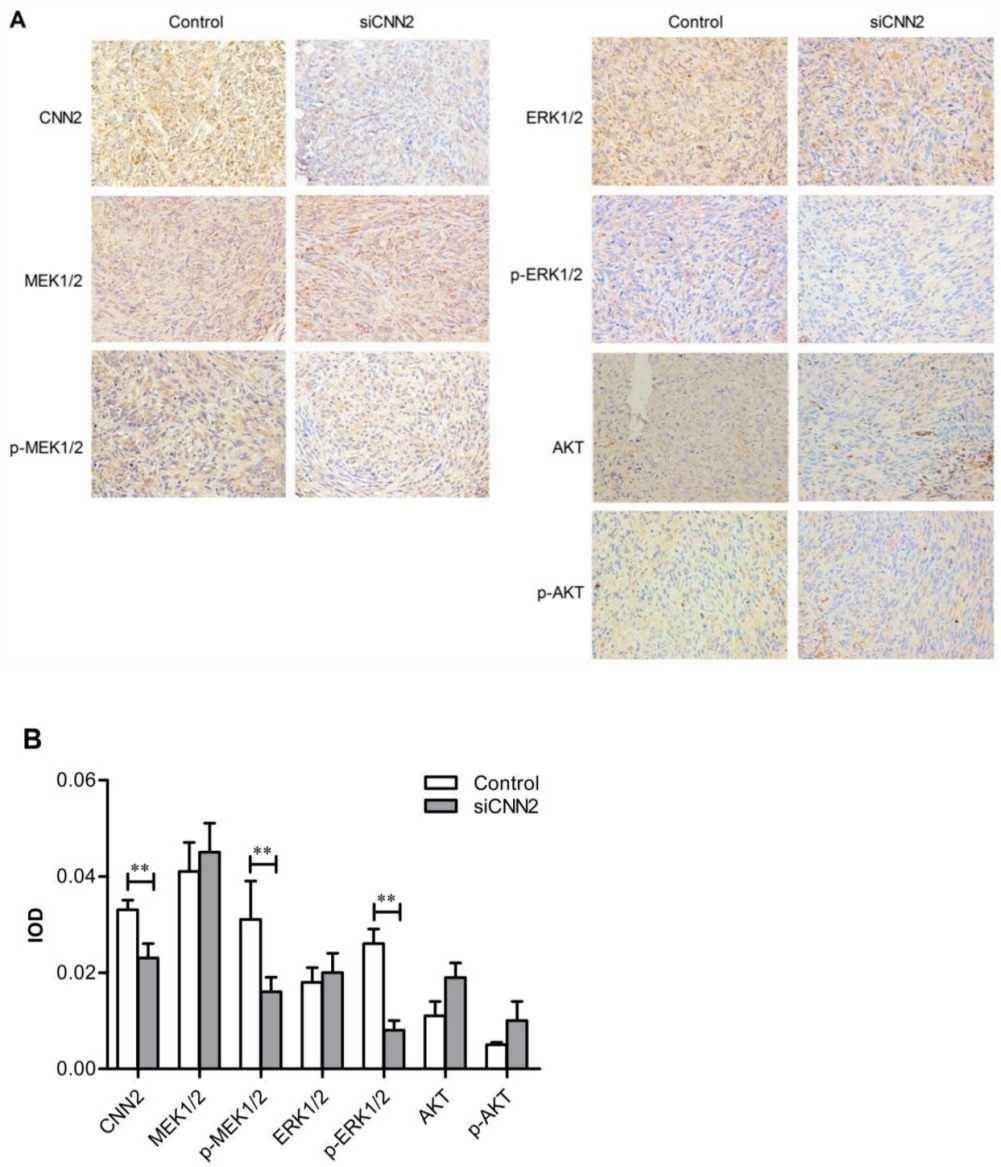
** Immunohistochemical results.** (A) Representative images from immunohistochemical analysis. (B) Integrated optical density of CNN2, MEK1/2, p-MEK1/2, ERK1/2, p-ERK1/2, AKT and p-AKT (^**^P < 0.01).

